# Longitudinal evaluation of olfactory function in individuals with Gaucher disease and *GBA1* mutation carriers with and without Parkinson's disease

**DOI:** 10.3389/fneur.2022.1039214

**Published:** 2022-10-18

**Authors:** Grisel J. Lopez, Jens Lichtenberg, Nahid Tayebi, Emory Ryan, Abigail L. Lecker, Ellen Sidransky

**Affiliations:** Medical Genetics Branch, National Human Genome Research Institute, National Institutes of Health, Bethesda, MD, United States

**Keywords:** Gaucher disease, *GBA1*, prodromal, Parkinson's, glucocerebrosidase

## Abstract

**Objective:**

Biallelic mutations in *GBA1*, which encodes the lysosomal enzyme glucocerebrosidase, cause the lysosomal storage disorder Gaucher disease (GD). In addition, mutations in *GBA1* are the most common genetic risk factor for future development of Parkinson's disease (PD). However, most mutation carriers will never develop parkinsonism. Olfactory dysfunction is often a prodromal symptom in patients with PD, appearing many years prior to motor dysfunction. The purpose of this study was to assess olfactory function longitudinally in individuals with and without parkinsonism who carry at least one *GBA1* mutation.

**Methods:**

One hundred seventeen individuals who participated in a natural history study of GD at the National Institutes of Health were evaluated using the University of Pennsylvania Smell Identification Test (UPSIT) during a 16-year period. Seventy patients with GD (13 with PD) and 47 *GBA1* carriers (9 with PD) were included. Fifty-six of the total (47.9%) were seen over multiple visits, and had UPSIT screening performed two to six times, with time intervals between testing ranging from 2 to 6 years. Comparative and control data were obtained from the Parkinson's Progression Markers Initiative (PPMI) database (519 individuals, including 340 with idiopathic PD and 179 healthy controls). Statistical analysis was performed using R.

**Results:**

Severe hyposmia and anosmia was evident in both *GBA1* heterozygotes and homozygotes with PD. 84% without parkinsonism had UPSIT scores >30, and those who underwent repeated testing maintained olfactory function over time. No statistically significant difference in UPSIT scores was found between mutation carriers with and without a family history of parkinsonism. A small group of individuals without PD scored in the moderate-severe microsmia range. No significant differences in olfaction were found among our *GBA1*-PD cohort and idiopathic PD cohort obtained from PPMI.

## Introduction

Olfactory dysfunction has been shown to increase in prevalence and severity with aging (1). However, it is a well-recognized prodromal non-motor symptom in neurodegenerative disorders, including Parkinson's disease (PD) (2), with a prevalence ranging from 45 to 90% in this population (3, 4). Hyposmia is also included as one of the supportive criteria in the revised Movement Disorder Society (MDS) clinical diagnostic criteria for Parkinson's disease (5). Assessment of odor identification in the PD population has become a routine part of most clinical research due to the ease of self-administration of the University of Pennsylvania Smell Identification Test (UPSIT), a “scratch-and-sniff” test that includes 40 microencapsulated odors with four alternative responses for each item (6). Because of the reported association between an increased risk of PD and hyposmia (7–9), evaluation of olfaction in genetically at-risk individuals could prove useful, as neuroprotective therapies continue to be an important focus in PD research (10).

The mechanism underlying olfactory dysfunction in PD has not been clearly elucidated. However, Lewy bodies, the pathological hallmark of PD that includes α-synuclein as a major constituent (11), have been reported in the olfactory bulb and can predict Lewy body pathology in other brain regions (12). In addition, neurotransmitter alterations have been associated with olfactory loss (4).

*GBA1* encodes for the lysosomal enzyme glucocerebrosidase. Individuals who carry biallelic (homozygous or compound heterozygous) mutations in this gene have the recessively inherited disorder Gaucher disease (GD). In addition, mutations in this gene are the most common genetic risk factor for the development of PD, with an overall odds ratio >5 for any *GBA1* mutation in patients with PD (13). Affected individuals with *GBA1*-associated PD have olfactory dysfunction (14, 15). In a previous study, when biallelic or heterozygous carriers of *GBA1* mutations without PD were followed and compared to healthy controls over a 4–5-year period, impaired olfaction was reported to be more pronounced in mutation carriers (16). This study evaluated olfactory function in a large cohort of patients with GD as well as heterozygous carriers, including those both with and without PD, during a 16-year interval.

## Materials and methods

### Participants

117 patients with GD and *GBA1* mutation carriers, with and without PD or family history of PD, were recruited at the National Institutes of Health from 2006 to 2022 under protocol NIH 86-HG-0096. In a rolling recruitment model, new participants were continuously enrolled in the study. Fifty-six participants (48%) were evaluated more than once during the 16-year period, ranging from one to five follow-up evaluations. At each visit, a physical and neurological evaluation, as well as an olfactory assessment using the UPSIT were performed. Of the individuals without PD, 33 of those with GD (57%) and 29 *GBA1* heterozygotes (76%) had a strong family history of PD. Selected data on 519 subjects were extracted from the Parkinson's Progression Markers Initiative (PPMI) database, including 340 subjects with idiopathic PD and 179 healthy controls.

### Genotyping

Genotyping of our cohort was performed using long template PCR amplification of the entire glucocerebrosidase gene, and Sanger sequencing was done as previously described (17). In particular we highlight pathological variants N370S (p.N409S) and L444P (p.L483P), while the complete set of genotypes is provided in Supplementary Figure 1.

### Data collection

Data were collected and managed using REDCap electronic data capture tools (18) hosted at the National Institutes of Health. Further comparative and control data on subjects with genetic PD, idiopathic PD, genetic unaffected PD cases, and healthy controls were obtained from the Parkinson's Progression Markers Initiative (PPMI) database (https://www.ppmi-info.org/accessdata-specimens/download-data). UPSIT scores were extracted from the meta data of the PPMI whole exome sequencing cohort.

### Statistical analysis

Collected data were evaluated using R version 4.2.0 (19) with the additional packages dplyr, ggplot2, ggrepel, tibble and tidyverse. Non-parametric tests (Wilcoxon rank sum test) were used to determine significant differences between participant subsets due to the data being non-normally distributed. The R scripts used to generate the different figures are provided in the Supplementary material.

## Results

### Cohort characteristics

Across the initial 117 individuals enrolled, 56 (47.9%) had GD, 38 (32.5%) were *GBA1* mutation carriers, and 23 (19.7%) were patients with GD or *GBA1* mutation carriers with PD. One subject was excluded because of a GD-unrelated E326K genotype, while two subjects, one from the GD cohort with PD (GD/PD) and one from the GD cohort with a family history of PD (GD/FH), were excluded because their UPSIT score fell in the “probable malingering” range (6, 20) (scores of 2 and 5), resulting in 114 actively evaluated individuals [49.12% (*n* = 56) with repeated visits].

Table 1 displays the specific groups and the total evaluations per group. The groups without PD were further divided into those with and without a first- or second-degree family member with PD (FH). 221 datapoints were generated, each representing the individual's UPSIT score at a given time point. As expected, individuals with PD showed the lowest UPSIT scores overall. The lowest mean UPSIT score for individuals without PD was for the GD/FH group (32.31) but did not reach statistical significance when compared to other non-PD groups. Sex distribution was predominantly male for the PD groups as well as the GD/FH group. No statistically significant differences in age distribution were seen among groups, although the PD groups were slightly older.

**Table 1:** Summary of cohort characteristics by groups.

**Groups**	**Tests per group**	**Age (mean, range)**	**Female (*N*,%)**	**Score (Max = 40) (mean, range)**
Total (*n =* 114)	221	57.09 (18–85)	111 (50%)	30.90 (9–40)
GC (*n =* 9)	13	46.69 (26–63)	8 (62%)	34.31 (28–39)
GC/FH (*n =* 28)	52	56.12 (27–85)	27 (52%)	33.54 (24–39)
GC/PD (*n =* 9)	15	60.73 (49–67)	6 (40%)	21.40 (9–38)
GD (*n =* 23)	37	54.59 (29–79)	31 (84%)	34.39 (24–39)
GD/FH (*n =* 32)	77	59.73 (18–80)	31 (40%)	32.31 (11–40)
GD/PD (*n =* 13)	27	57.85 (37–74)	8 (30%)	20.48 (10–34)

A total of 221 UPSIT evaluation were conducted for 114 individuals with different disease presentations (GC, *GBA1* carriers; FH, family history; GD, Gaucher disease; PD, Parkinson's disease). A breakdown of age, sex and UPSIT score associated with each disease group is presented here.

**Figure 1:**
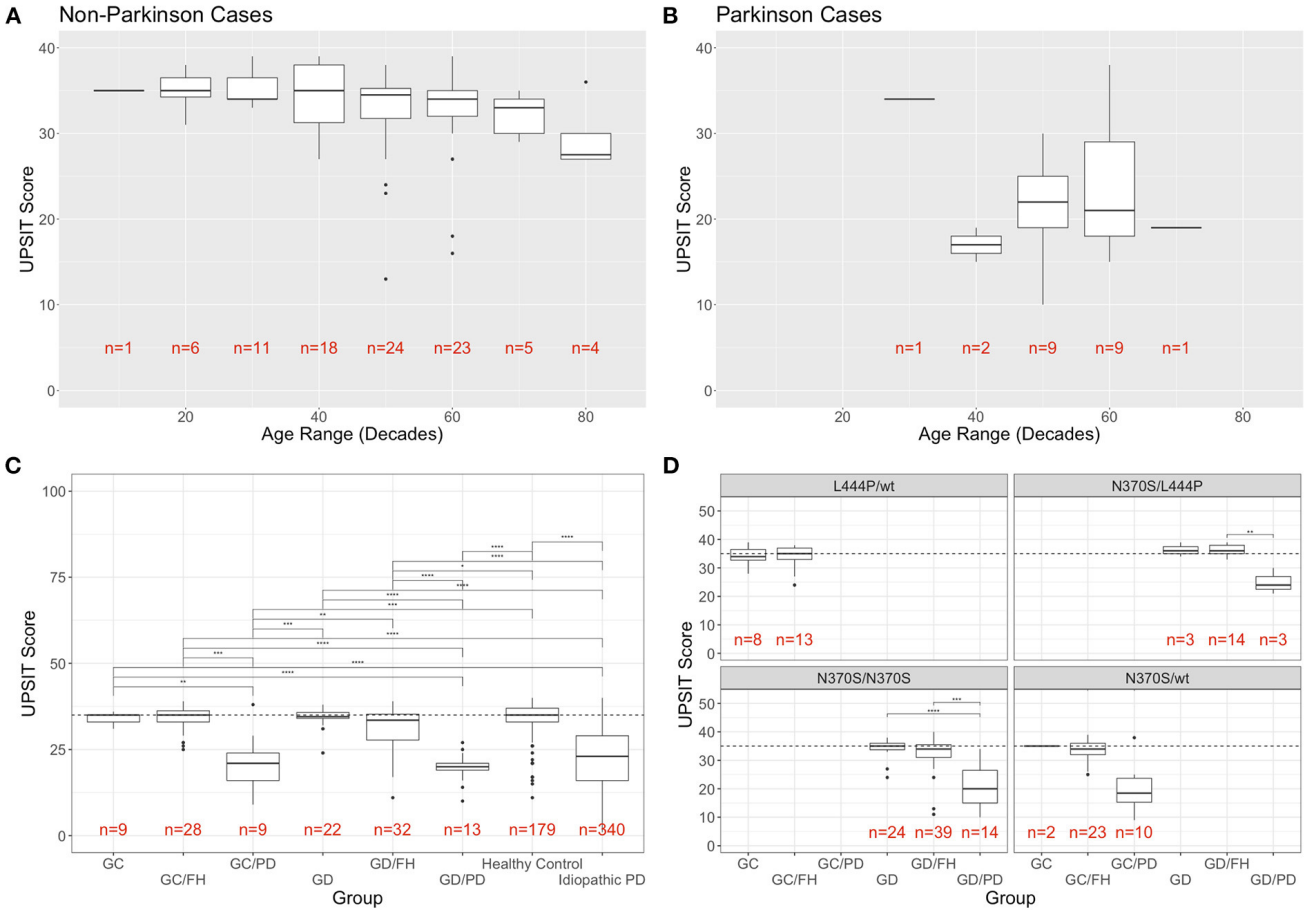
Distribution of UPSIT scores by age, group, and genotype. **(A)** Individuals without PD decline in olfactory identification scores (taken at the initial visit) with increasing age, while this trend was not observed in patients with PD **(B). (C)** Mean UPSIT scores at the most recent visit are shown for each group (GC, *GBA1* carriers; FH, family history; GD, Gaucher disease; PD, Parkinson's disease). For comparison, the data from the cases of idiopathic PD and controls from the PPMI cohort is included in the far right. **(D)** Breakdown of UPSIT scores by disease group and most frequent genotypes. Significance is determined using Wilcoxon rank sum test (*p*-values: ^**^ <0.01; ^***^ <0.001; ^****^ <0.0001).

The groups without PD showed a mild decline in olfaction by age at evaluation (age range in decades), as previously reported in healthy individuals (6) (Figure 1A). This was especially true of subjects in their eighth and ninth decade. When analyzing the scores for PD cases for the same age ranges, higher UPSIT scores were seen with increased age at the initial visit for groups with *n* > 1 (Figure 1B).

When comparing the UPSIT scores for each group, using the most recent visit for individuals with repeated testing (Figure 1C), the only statistically significant differences were between the groups with and without PD. No significant difference was conferred by family history of PD, heterozygosity, or homozygosity. There were seven individuals in the groups without PD who were classified as outliers in their respective groups, demonstrating the most severe olfactory deficit. When comparing the NIH cohort data with the UPSIT scores of 519 individuals extracted from the PPMI whole exome sequencing meta data, no statistically significant differences between our heterozygous or homozygous *GBA1*-PD and those with idiopathic PD (*n* = 340) was found, while the healthy controls (*n* = 179) differed significantly from our PD groups. The UPSIT scores of our *GBA1* heterozygotes or homozygotes without PD also differed significantly when compared to the idiopathic PD groups from PPMI. While carriers with and without family history as well as GD patients without a family history showed no difference to PPMI healthy controls, GD patients with a family history were significantly different from healthy controls (Figure 1C).

Analysis by group and most frequent genotypes in this cohort is shown in Figure 1D. A breakdown of all genotypes found in the cohort and the number of participants that are associated with them is provided in Supplementary Figure 1.

No phenotype/genotype correlations were found with regards to olfactory function in subjects without PD, including between those with and without a family history of PD. While the general trend of decreased UPSIT scores in PD groups compared to non-PD groups remains consistent for each genotype, due to the limited sample size the only statistically significant difference was seen specifically in N370S homozygotes between those without PD and without FH (GD) and those with PD (Figure 1D). Genotypes found in only one individual were not included in this analysis.

### Longitudinal characteristics

**Figure 2:**
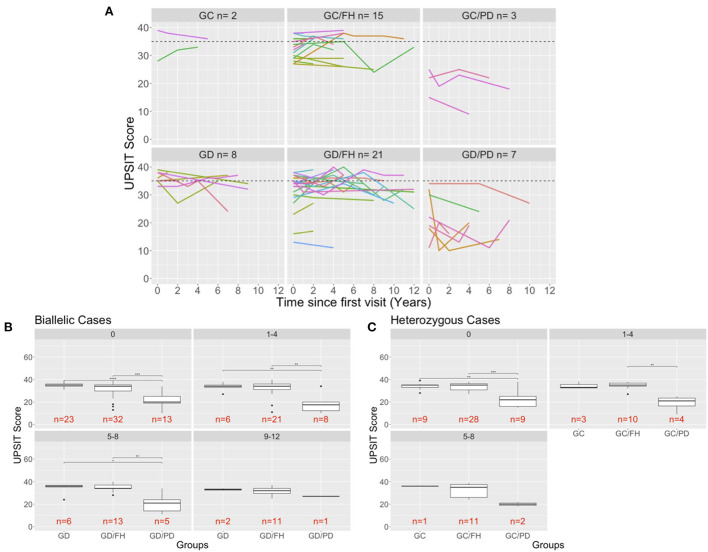
Longitudinal evaluation of olfaction. **(A)** The UPSIT scores of individuals that were evaluated multiple time in the clinic are shown and each test date is shown in relation to the time of that has passed since the individuals first visit to the clinic. A dash line is drawn at 35 representing healthy UPSIT scores. **(B,C)** Comparison of biallelic **(B)** and heterozygous **(C)** groups across the different age ranges (0: baseline visit for a patient, 1–4: visits up to 4 years after the baseline visit). Significance is determined using Wilcoxon rank sum test (*p*-values: ^**^ <0.01; ^***^ <0.001; ^****^ <0.0001). The 9–12-year age for the heterozygous groups was removed because only carriers with a family history of PD were measured that far out from the first visit.

Forty-nine percent of participants in our study had up to six tests, repeated at different time intervals ranging from 2 to 6 years. Figure 2A shows UPSIT scores in relation to time since the initial olfactory evaluation. For the majority of patients with PD, hyposmia/anosmia was identified at their initial visit, followed by a steady decline over time. For the individuals without PD, some variability was noted in scores per individual with repeated testing, although scores tended to remain stable over time, similar to what has been reported in normal cohorts (6).

To better assess and compare the longitudinal results by group, the scores were categorized into intervals of 4 years since the first visit for individuals with biallelic mutations (Figure 2B) and heterozygous carriers (Figure 2C). No significant differences were found between the groups without PD, although there was a slight decline in UPSIT scores with age. This is in contrast to the PD groups, which demonstrated a decline at each time interval (Figures 2B,C). The small number of cases prohibited an analysis for the 9–12 years interval in the heterozygous carriers.

## Discussion

This longitudinal olfactory assessment in subjects with *GBA1* mutations over a 16-year period is the largest and longest to date, following both homozygotes and heterozygotes including a large number with a family history of parkinsonism. As expected, patients with PD, regardless of whether they carried biallelic or heterozygous mutations, showed abnormal olfactory function. Most individuals without PD have maintained stable olfactory scores over time, although a subgroup of subjects initially scored in the low normal/mild microsmia range, suggesting possible baseline olfactory impairment in mutation carriers, described in previous studies (16). The reason for the low baseline scores merits further investigation and may result from other unrelated factors. In addition, a few individuals with severe microsmia/anosmia without PD reported plausible non-parkinsonian explanations for their deficit such as a chronic nasal canula due to oxygen dependance, onset directly following a viral infection 40 years prior to testing, and multiple surgical resections of nasal polyps, among others, highlighting the need for additional medical history when interpreting absolute scores. This is especially true with regard to recent infection with SARS-CoV-2 as the virus can lead to impaired olfaction (21). Two individuals scored in the “probable malingering” (6, 20) range and were excluded as they skewed the distribution of scores. Seven individuals in the non-PD groups had scores that fell far from the other data points within their group (outliers). However, when looking closer at the individuals in this category, five were between the ages 74–83 and one was age 60 but had multiple nasal surgeries, accounting for the likely etiology of the hyposmia.

Comparison with the PPMI data demonstrates that among all subjects with PD, hyposmia was prevalent, but *GBA1* mutations did not confer additional modulation to odor identification. It is important to emphasize that none of the at-risk individuals in this cohort have developed parkinsonism over the course of the study. The lack of individuals developing parkinsonism (converters) in the cohort, prohibits a conclusive interpretation of UPSIT scores in subjects with *GBA1* biallelic or heterozygous mutations but without PD. It is important to note that due to the nature of the *GBA1* locus and its sequence homology to pseudogene *GBAP*, it is possible that *GBA1* mutations may be included in the idiopathic PD groups although the number of cases would likely be small and not impact the statistical comparisons.

Both the advanced age in the majority of outliers in our study and the variability in scores over time that is seen even in healthy individuals (6), are further indications of the limitations of odor identification scores as an early biomarker for parkinsonism. Clearly, additional parameters such as REM behavior disorder, autonomic dysfunction, and/or neuroimaging, as well as yet to be defined biomarkers will be needed in combination with or instead of olfactory screening, when trying to identify individuals at highest risk.

Despite being one of the largest and longest running studies assessing odor identification in *GBA1* subjects, the cohort still represents a small sample size, limiting rigorous statistical analysis. This limitation is even more apparent when focusing on genotype correlations and time between recurrent visits.

Continued evaluation of individuals with abnormal scores not explained by their medical history and a comparison against longitudinal assessments for an aging healthy cohort is necessary to determine whether deficits observed at this time are identifying those on a trajectory to develop parkinsonism in the future. This extended follow-up will also help determine if the rate or pattern of decline in olfactory function might eventually prove useful as a disease biomarker. The study confirms the observed low penetrance of parkinsonism among *GBA1* mutation carriers (22, 23). Thus, a focus on the identification of additional biomarkers is needed to predict those *GBA1* mutation carriers most likely to develop PD who might benefit from future neuroprotective therapies.

## Data availability statement

The original contributions presented in the study are included in the article/Supplementary material, further inquiries can be directed to the corresponding author/s.

## Ethics statement

The studies involving human participants were reviewed and approved by Institutional Review Board of the National Institutes of Health. The patients/participants provided their written informed consent to participate in this study.

## Author contributions

GL, ER, and ES collected the data. GL, JL, and AL conducted the data analysis. NT performed the *GBA1* sequencing. GL, JL, and ES wrote the manuscript. All authors contributed to the article and approved the submitted version.

## Funding

This work was supported by the intramural research programs of the National Human Genome Research Institute and the National Institutes of Health. PPMI—a public–private partnership is funded by The Michael J. Fox Foundation for Parkinson's Research and funding partners.

## Conflict of interest

The authors declare that the research was conducted in the absence of any commercial or financial relationships that could be construed as a potential conflict of interest.

## Publisher's note

All claims expressed in this article are solely those of the authors and do not necessarily represent those of their affiliated organizations, or those of the publisher, the editors and the reviewers. Any product that may be evaluated in this article, or claim that may be made by its manufacturer, is not guaranteed or endorsed by the publisher.

## References

[1] Attems J Walker L Jellinger KA. Olfaction and aging: a mini-review. Gerontology. (2015) 61:485–90. 10.1159/000381619 25968962

[2] Marin C Vilas D Langdon C Alobid I López-Chacón M Haehner A. Olfactory dysfunction in neurodegenerative diseases. Curr Allergy Asthma Rep. (2018) 18:42. 10.1007/s11882-018-0796-4 29904888

[3] Ansari KA Johnson A. Olfactory function in patients with Parkinson's disease. J Chronic Dis. (1975) 28:493–7. 10.1016/0021-9681(75)90058-2 1176578

[4] Doty RL. Olfactory dysfunction in Parkinson disease. Nat Rev Neurol. (2012) 8:329–39. 10.1038/nrneurol.2012.80 22584158

[5] Postuma RB Berg D Stern M Poewe W Olanow CW Oertel W. MDS clinical diagnostic criteria for Parkinson's disease. Mov Disord. (2015) 30:1591–601. 10.1002/mds.26424 26474316

[6] Doty RL Shaman P Kimmelman CP Dann MS. University of Pennsylvania Smell Identification Test: a rapid quantitative olfactory function test for the clinic. Laryngoscope. (1984) 94:176–8. 10.1288/00005537-198402000-00004 6694486

[7] Ponsen MM Stoffers D Twisk JW Wolters E Berendse HW. Hyposmia and executive dysfunction as predictors of future Parkinson's disease: a prospective study. Mov Disord. (2009) 24:1060–5. 10.1002/mds.22534 19353591

[8] Reichmann H. Premotor diagnosis of Parkinson's disease. Neurosci Bull. (2017) 33:526–34. 10.1007/s12264-017-0159-5 28776303 PMC5636732

[9] Driver-Dunckley E Adler CH Hentz JG Dugger BN Shill HA Caviness JN. Olfactory dysfunction in incidental Lewy body disease and Parkinson's disease. Parkinsonism Relat Disord. (2014) 20:1260–2. 10.1016/j.parkreldis.2014.08.006 25172126 PMC4835172

[10] Salamon A Zádori D Szpisjak L Klivényi P Vécsei L. Neuroprotection in Parkinson's disease: facts and hopes. J Neural Transm (Vienna). (2020) 127:821–9. 10.1007/s00702-019-02115-8 31828513 PMC7242234

[11] Spillantini MG Schmidt ML Lee VM Trojanowski JQ Jakes R Goedert M. Alpha-synuclein in Lewy bodies. Nature. (1997) 388:839–40. 10.1038/42166 9278044

[12] Beach TG White CL III Hladik CL Sabbagh MN Connor DJ Shill HA. Olfactory bulb alpha-synucleinopathy has high specificity and sensitivity for Lewy body disorders. Acta Neuropathol. (2009) 117:169–74. 10.1007/s00401-008-0450-7 18982334 PMC2631085

[13] Sidransky E Nalls MA Aasly JO Aharon-Peretz J Annesi G Barbosa ER. Multicenter analysis of glucocerebrosidase mutations in Parkinson's disease. N Engl J Med. (2009) 361:1651–61. 10.1056/NEJMoa0901281 19846850 PMC2856322

[14] Goker-Alpan O Lopez G Vithayathil J Davis J Hallett M Sidransky E. The spectrum of parkinsonian manifestations associated with glucocerebrosidase mutations. Arch Neurol. (2008) 65:1353–7. 10.1001/archneur.65.10.1353 18852351 PMC2629407

[15] Avenali M Toffoli M Mullin S McNeil A Hughes DA Mehta A. Evolution of prodromal parkinsonian features in a cohort of GBA mutation-positive individuals: a 6-year longitudinal study. J Neurol Neurosurg Psychiatry. (2019) 90:1091–7. 10.1136/jnnp-2019-320394 31221723

[16] Mullin S Beavan M Bestwick J McNeill A Proukakis C Cox T. Evolution and clustering of prodromal parkinsonian features in GBA1 carriers. Mov Disord. (2019) 34:1365–73. 10.1002/mds.27775 31251436 PMC6790937

[17] Stone DL Tayebi N Orvisky E Stubblefield B Madike V Sidransky E. Glucocerebrosidase gene mutations in patients with type 2 Gaucher disease. Hum Mutat. (2000) 15:181–8. 10.1002/(SICI)1098-1004(200002)15:2<181::AIDHUMU7>3.0.CO;2-S 10649495

[18] Harris PA Taylor R Minor BL Elliott V Fernandez M O'Neal L. The REDCap consortium: building an international community of software platform partners. J Biomed Inform. (2019) 95:103208. 10.1016/j.jbi.2019.103208 31078660 PMC7254481

[19] Giorgi FM Ceraolo C Mercatelli D. The R language: an engine for bioinformatics and data science. Life. (2022) 12:648. 10.3390/life12050648 35629316 PMC9148156

[20] Kurtz DB White TL Hornung DE Belknap E. What a tangled web we weave: discriminating between malingering and anosmia. Chem Senses. (1999) 24:697–700. 10.1093/chemse/24.6.697 10587503

[21] Moein ST Hashemian SM Mansourafshar B Khorram-Tousi A Tabarsi P Doty RL. Smell dysfunction: a biomarker for COVID-19. Int Forum Allergy Rhinol. (2020) 10:944–50. 10.1002/alr.22587 32301284 PMC7262123

[22] Anheim M Elbaz A Lesage S Durr A Condroyer C Viallet F. Penetrance of Parkinson disease in glucocerebrosidase gene mutation carriers. Neurology. (2012) 78:417–20. 10.1212/WNL.0b013e318245f476 22282650

[23] Rana HQ Balwani M Bier L Alcalay RN. Age-specific Parkinson disease risk in GBA mutation carriers: information for genetic counseling. Genet Med. (2013) 15:146–9. 10.1038/gim.2012.107 22935721 PMC3519952

