# Supplementary material for: Longitudinal evaluation of olfactory function in individuals with Gaucher disease and GBA1 mutation carriers with and without Parkinson's disease
Source: Front Neurol. 2022 Oct 18;13:1039214. doi: 10.3389/fneur.2022.1039214 (PMC9622935; doi:10.3389/fneur.2022.1039214)
Supplement: Supplementary file 1 [file Data_Sheet_1.DOCX]

# Supplement

## R Scripts

### Figure 1A

library(ggplot2)

library(ggrepel)

library(tibble)

data=read.csv("~/Box/Lab_Notebook/Grisel_DATASET/AbbySetNoName_06242022_withUID_outliersRemoved_deltaUPSIT.csv",header=TRUE,sep=",")

newdata<-data[((data$Group=="GD" | data$Group=="GC" | data$Group=="GD/FH" | data$Group=="GC/FH") & data$Genotype!="E326K/wt" & data$VisitNumber==0),]

ggplot(newdata, aes(x=AgeBin, y=Total.1)) + geom_boxplot(aes(group=AgeBin)) + labs(x="Age Range (Decades)",y="UPSIT Score", title="Non-Parkinson Cases") + geom_text(aes(label=gsub(" ", "",paste("n=",..count..))), y=5, stat='count', colour="red", size=8) + coord_cartesian(ylim=c(0,40),xlim=c(5,85)) + theme(text = element_text(size = 25))

### Figure 1B

library(ggplot2)

library(ggrepel)

library(tibble)

data=read.csv("~/Box/Lab_Notebook/Grisel_DATASET/AbbySetNoName_06242022_withUID_outliersRemoved_deltaUPSIT.csv",header=TRUE,sep=",")

newdata<-data[((data$Group=="GD/PD" | data$Group=="GC/PD") & data$VisitNumber==0),]

ggplot(newdata, aes(x=AgeBin, y=Total.1)) + geom_boxplot(aes(group=AgeBin)) + labs(x="Age Range (Decades)",y="UPSIT Score", title="Parkinson Cases") + geom_text(aes(label=gsub(" ","",paste("n=",..count..))), y=5, stat='count', colour="red", size=8) + coord_cartesian(ylim=c(0,40),xlim=c(5,85)) + theme(text = element_text(size = 25))

### Figure 1C

library(ggplot2)

library(ggpubr)

library(tidyverse)

data=read.csv("~/OneDrive - National Institutes of Health/Lab_Notebook/Sidransky_Ongoing/Grisel_06242022_withUID_outliersRemoved_deltaUPSIT_PPMI.csv",header=TRUE,sep=",")

newdata<-data[(data$Group!="?/PD" & data$Group!="GD/ND" & data$Group!="WT/FH" & data$Group!="Conv" & data$Group!="GD3/PD" & data$Group!= "WT" & data$Group!="Genetic PD" & data$Group!="Genetic Unaffected" & data$Group!="SWEDD" & data$Group!="Other ND" & data$Genotype!="E326K/wt" & (data$LastVisit==TRUE | data$LastVisit=="")),]

stat_pvalue <- newdata %>% rstatix::wilcox_test(Total.1 ~ Group) %>% filter(p < 0.05) %>% rstatix::add_significance("p") %>% rstatix::add_y_position() %>% mutate(y.position = seq(min(y.position), max(y.position),length.out = n()))

ggplot(newdata, aes(x=Group, y=Total.1)) + geom_boxplot() + ggpubr::stat_pvalue_manual(stat_pvalue, label = "p.signif") + theme_bw(base_size = 16) + labs(x="Group",y="UPSIT Score") + geom_text(aes(label= gsub(" ","",paste("n=",..count..))), y=2, stat='count', colour="red", size=8) + geom_hline(yintercept=35,linetype='dashed') + coord_cartesian(ylim=c(0,100)) + scale_x_discrete(guide=guide_axis(n.dodge = 2)) + theme(text = element_text(size = 25))

### Figure 1D

library(ggplot2)

library(ggpubr)

library(tidyverse)

data=read.csv("~/OneDrive - National Institutes of Health/Lab_Notebook/Sidransky_Ongoing/Grisel_06242022_withUID_outliersRemoved_deltaUPSIT_PPMI.csv",header=TRUE,sep=",")

newdata<-data[(data$Group!="?/PD" & data$Group!="GD/ND" & data$Group!="WT/FH" & data$Group!="Conv" & data$Group!="GD3/PD" & data$Group!="Healthy Control" & data$Group!="SWEDD" & data$Group!="Other ND" & (data$Genotype=="N370S/N370S" | data$Genotype=="N370S/L444P" | data$Genotype=="N370S/wt" | data$Genotype=="L444P/wt")),]

stat_pvalue <- newdata %>% group_by(Genotype) %>% rstatix::wilcox_test(Total.1 ~ Group) %>% rstatix::add_significance("p") %>% rstatix::add_y_position() %>% mutate(y.position = seq(min(y.position), max(y.position),length.out = n()))

ggplot(newdata, aes(x=Group, y=Total.1)) + geom_boxplot() +facet_wrap(.~Genotype,ncol=2)+ ggpubr::stat_pvalue_manual(stat_pvalue, hide.ns=TRUE, step.increase=0.1, label = "p.signif") + theme_bw(base_size = 16) + geom_hline(yintercept=35,linetype='dashed') + labs(x="Group",y="UPSIT Score")+ scale_y_continuous(expand = expansion(mult = c(0.05, 0.1))) + geom_text(aes(label=gsub(" ","",paste("n=",..count..))), y=5, stat='count', colour="red", size=8) + coord_cartesian(ylim=c(0,50)) + scale_x_discrete(guide=guide_axis(n.dodge = 2)) + theme(text = element_text(size = 25))

### Figure 2A

library(ggplot2)

library(ggrepel)

library(tibble)

library(dplyr)

data=read.csv("~/Box/Lab_Notebook/Grisel_DATASET/AbbySetNoName_06242022_withUID_outliersRemoved_deltaUPSIT.csv",header=TRUE,sep=",")

dataclean <-data[(data$Group!="?/PD" & data$Group!="GD/ND" & data$Group!="WT/FH" & data$Group!="Conv" & data$Group!="GD3/PD" & data$Group!="WT"),]

is_outlier <- function(x) {

return(x < quantile(x, 0.25,na.rm= TRUE) - 1.5 * IQR(x,na.rm=TRUE) | x > quantile(x, 0.75,na.rm= TRUE) + 1.5 * IQR(x,na.rm=TRUE))

}

datamod <- dataclean %>% tibble::rownames_to_column(var="outlier") %>% group_by(Since.First.Years,Group) %>% mutate(is_outlier=ifelse(is_outlier(Total.1), Total.1, as.numeric(NA)))

datamod$outlier[which(is.na(datamod$is_outlier))] <- as.numeric(NA)

datamod = datamod %>% mutate(OUT_UID=ifelse(!(is.na(outlier)),SubjectID,NA))

datamod2<-subset(datamod,(datamod$Total.1!=""))

labeling <- datamod2 %>% group_by(Group) %>% summarize(n=length(which(table(SubjectID)>1)))

hum_names <- as_labeller(function(x) paste(x,'n=', labeling[labeling $Group==x,]$n))

datamod2 %>% group_by(Group) %>% mutate(dummy_var = as.character(x = factor(x = as.factor(SubjectID),labels = seq_len(length.out = n_distinct(x = as.factor(SubjectID)))))) %>% ungroup() %>% ggplot(mapping = aes(x = Since.First.Years, y = Total.1)) + geom_line(mapping = aes(colour = dummy_var),size=1) + facet_wrap(. ~ Group, labeller=hum_names) + scale_color_discrete(guide = "none") + labs(x="Time since first visit (Years)",y="UPSIT Score") + geom_hline(yintercept=35,linetype='dashed') + scale_x_continuous(n.breaks=7,labels=c(0,2,4,6,8,10,12)) + coord_cartesian(ylim=c(0,40)) + theme(text = element_text(size = 25))

### Figure 2B

library(ggplot2)

library(ggrepel)

library(tibble)

library(dplyr)

data=read.csv("~/Box/Lab_Notebook/Grisel_DATASET/AbbySetNoName_06242022_withUID_outliersRemoved_deltaUPSIT.csv",header=TRUE,sep=",")

newdata<-data[(data$Group!="?/PD" & data$Group!="GD/ND" & data$Group!="WT/FH" & data$Group!="Conv" & data$Group!="GD3/PD" & data$Group!="WT" & data$Group!="GC" & data$Group!="GC/PD" & data$Group!="GC/FH"),]

stat_pvalue <- newdata %>% group_by(Since.Binned) %>% rstatix::wilcox_test(Total.1 ~ Group) %>% rstatix::add_significance("p") %>% rstatix::add_y_position() %>% mutate(y.position = seq(min(y.position), max(y.position),length.out = n()))

labeling <- newdata %>% group_by(Since.Binned) %>% tally()

hum_names <- as_labeller(function(x) paste(x,'n=', labeling[labeling $Group==x,]$n))

ggplot(newdata, aes(x=Group, y=Total.1)) + geom_boxplot() + ggpubr::stat_pvalue_manual(stat_pvalue, hide.ns=TRUE, step.increase=0.1, label = "p.signif") + facet_wrap(. ~ Since.Binned, ncol=2) + geom_text(aes(label=gsub(" ","",paste("n=",..count..))), y=5, stat='count', colour="red", size=8) + coord_cartesian(ylim=c(0,72)) + labs(x="Groups",y="UPSIT Score",title="Biallelic Cases") + theme(text = element_text(size = 25))

### Figure 2C

library(ggplot2)

library(ggrepel)

library(tibble)

library(dplyr)

data=read.csv("~/Box/Lab_Notebook/Grisel_DATASET/AbbySetNoName_06242022_withUID_outliersRemoved_deltaUPSIT.csv",header=TRUE,sep=",")

newdata<-data[(data$Group!="?/PD" & data$Group!="GD/ND" & data$Group!="WT/FH" & data$Group!="Conv" & data$Group!="GD3/PD" & data$Group!="WT" & data$Group!="GD" & data$Group!="GD/PD" & data$Group!="GD/FH" & data$Since.Binned!="9-12" & data$Genotype!="E326K/wt"),]

stat_pvalue <- newdata %>% group_by(Since.Binned) %>% rstatix::wilcox_test(Total.1 ~ Group) %>% rstatix::add_significance("p") %>% rstatix::add_y_position() %>% mutate(y.position = seq(min(y.position), max(y.position),length.out = n()))

labeling <- newdata %>% group_by(Since.Binned) %>% tally()

hum_names <- as_labeller(function(x) paste(x,'n=', labeling[labeling $Group==x,]$n))

ggplot(newdata, aes(x=Group, y=Total.1)) + geom_boxplot() + ggpubr::stat_pvalue_manual(stat_pvalue, hide.ns=TRUE, step.increase=0.1, label = "p.signif") + facet_wrap(. ~ Since.Binned, ncol=2) + geom_text(aes(label=gsub(" ","",paste("n=",..count..))), y=5, stat='count', colour="red", size=8) + coord_cartesian(ylim=c(0,72)) + labs(x="Groups",y="UPSIT Score",title="Heterozygous Cases") + theme(text = element_text(size = 25))

### Supplemental Figure 1

library(ggplot2)

library(ggpubr)

library(tidyverse)

data=read.csv("~/OneDrive - National Institutes of Health/Lab_Notebook/Sidransky_Ongoing/Grisel_06242022_withUID_outliersRemoved_deltaUPSIT_PPMI.csv",header=TRUE,sep=",")

newdata<-data[(data$Group!="?/PD" & data$Group!="GD/ND" & data$Group!="WT/FH" & data$Group!="Conv" & data$Group!="GD3/PD" & data$Group!="Healthy Control" & data$Group!="SWEDD" & data$Group!="Other ND" & data$Group!="Genetic Unaffected" & data$Group!="Idiopathic PD" & data$Group!="Genetic PD" & data$Group!="WT" & data$Genotype!="" & data$Genotype!="E326K/wt"),]

ggplot(newdata, aes(x=Group, y=Total.1)) + geom_boxplot() +facet_wrap(.~Genotype,ncol=4) + theme_bw(base_size = 16) + geom_hline(yintercept=35,linetype='dashed') + labs(x="Group",y="UPSIT Score")+ scale_y_continuous(expand = expansion(mult = c(0.05, 0.1))) + geom_text(aes(label=gsub(" ","",paste("n=",..count..))), y=5, stat='count', colour="red", size=8) + coord_cartesian(ylim=c(0,50)) + scale_x_discrete(guide=guide_axis(n.dodge = 2)) + theme(text = element_text(size = 20))

## Supplemental Figure 1


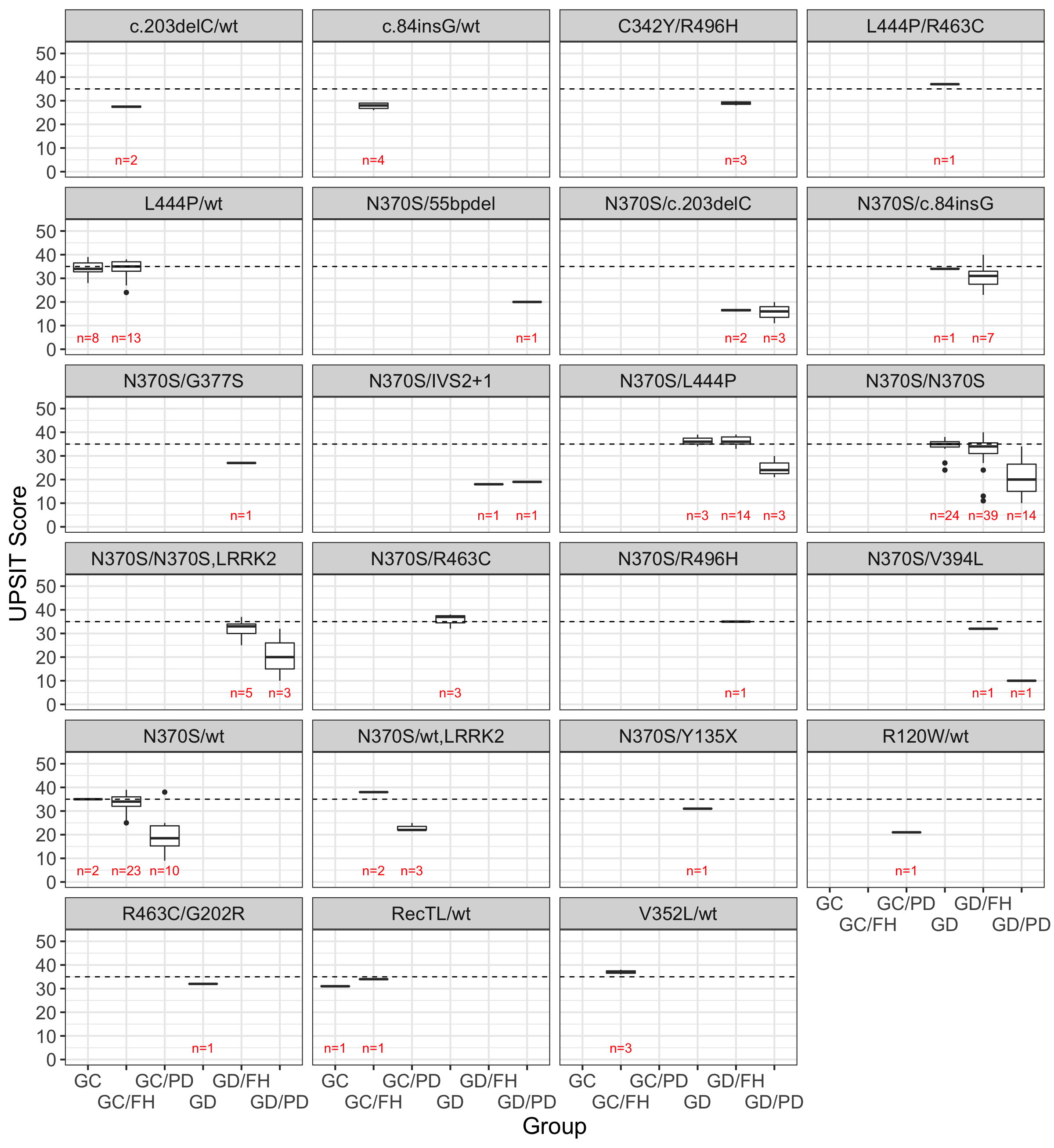


Supplemental Figure 1. Breakdown of UPSIT scores in the observed genotypes in the cohort across the different groups.
