# Supplementary figures and images for: Longitudinal evaluation of olfactory function in individuals with Gaucher disease and GBA1 mutation carriers with and without Parkinson's disease
Source: Front Neurol. 2022 Oct 18;13:1039214. doi: 10.3389/fneur.2022.1039214 (PMC9622935; doi:10.3389/fneur.2022.1039214)

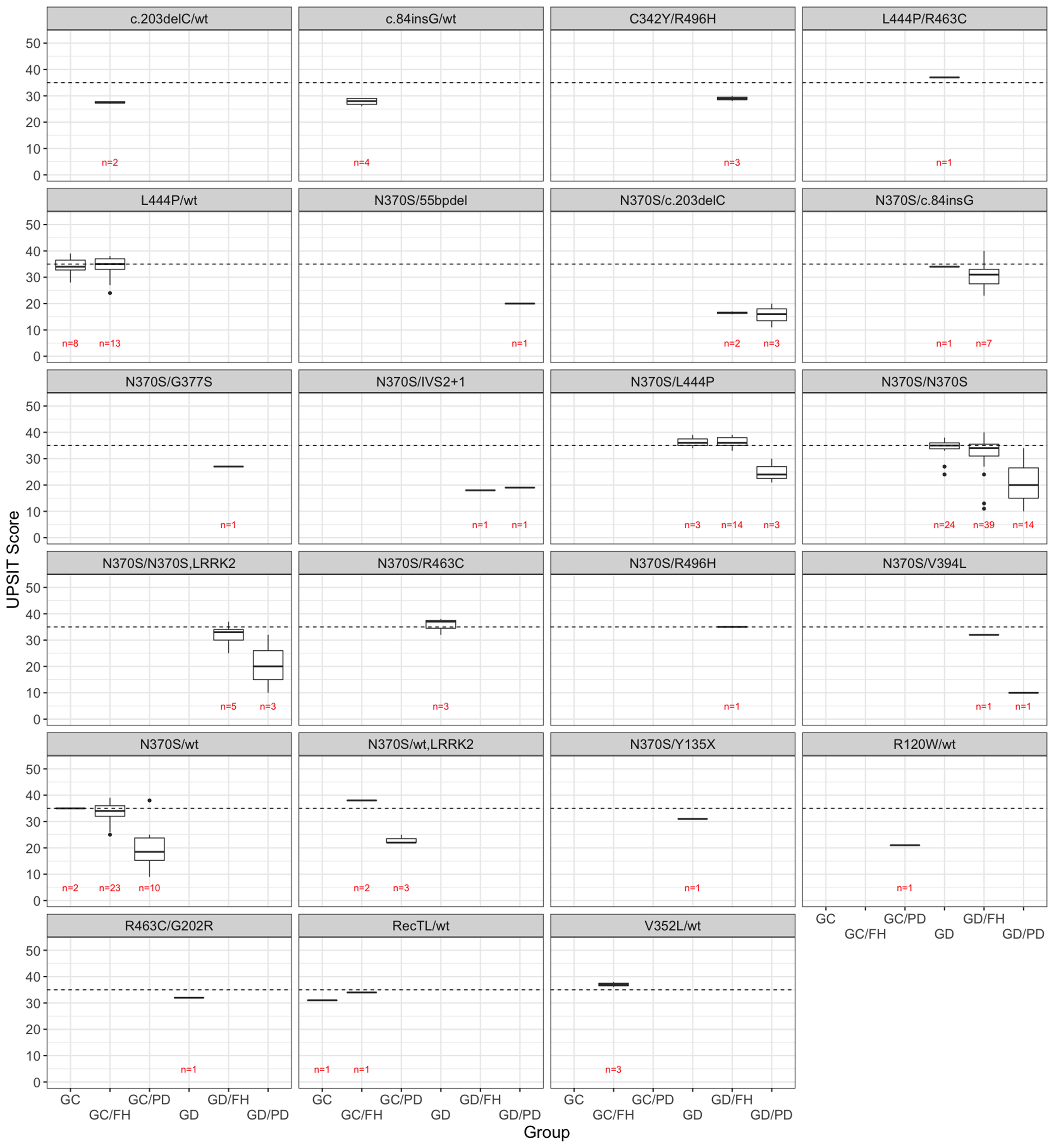

Supplement: Supplementary file 2 [file Image_1.TIF]
